# Postharvest Monitoring of Tomato Ripening Using the Dynamic Laser Speckle

**DOI:** 10.3390/s18041093

**Published:** 2018-04-04

**Authors:** Piotr Mariusz Pieczywek, Małgorzata Nowacka, Magdalena Dadan, Artur Wiktor, Katarzyna Rybak, Dorota Witrowa-Rajchert, Artur Zdunek

**Affiliations:** 1 Institute of Agrophysics, Polish Academy of Sciences, Doświadczalna 4, 20-290 Lublin, Poland; a.zdunek@ipan.lublin.pl; 2 Department of Food Engineering and Process Management, Faculty of Food Sciences, Warsaw University of Life Sciences (SGGW), Nowoursynowska 159c, 02-776 Warsaw, Poland; malgorzata_rzaca@sggw.pl (M.N.); magdalena_dadan@sggw.pl (M.D.); artur_wiktor@sggw.pl (A.W.); katarzyna_rybak@sggw.pl (K.R.); dorota_witrowa_rajchert@sggw.pl (D.W.-R.)

**Keywords:** biospeckle, optical sensor, video processing, tomato, maturation, shelf life, postharvest quality

## Abstract

The dynamic laser speckle (biospeckle) method was tested as a potential tool for the assessment and monitoring of the maturity stage of tomatoes. Two tomato cultivars—Admiro and Starbuck—were tested. The process of climacteric maturation of tomatoes was monitored during a shelf life storage experiment. The biospeckle phenomena were captured using 640 nm and 830 nm laser light wavelength, and analysed using two activity descriptors based on biospeckle pattern decorrelation—C4 and ε. The well-established optical parameters of tomatoes skin were used as a reference method (luminosity, a*/b*, chroma). Both methods were tested with respect to their prediction capabilities of the maturity and destructive indicators of tomatoes—firmness, chlorophyll and carotenoids content. The statistical significance of the tested relationships were investigated by means of linear regression models. The climacteric maturation of tomato fruit was associated with an increase in biospckle activity. Compared to the 830 nm laser wavelength the biospeckle activity measured at 640 nm enabled more accurate predictions of firmness, chlorophyll and carotenoids content. At 640 nm laser wavelength both activity descriptors (C4 and ε) provided similar results, while at 830 nm the ε showed slightly better performance. The linear regression models showed that biospeckle activity descriptors had a higher correlation with chlorophyll and carotenoids content than the a*/b* ratio and luminosity. The results for chroma were comparable with the results for both biospeckle activity indicators. The biospeckle method showed very good results in terms of maturation monitoring and the prediction of the maturity indices of tomatoes, proving the possibility of practical implementation of this method for the determination of the maturity stage of tomatoes.

## 1. Introduction

Tomato is a climacteric fruit, with a relatively short postharvest life determined by ethylene [1]. As for many other climacteric fruit and vegetables ripeness assessment of the agricultural crop ensures optimal harvest time, which has a huge impact on the postharvest quality of the yield. Harvesting at the full ripening stage results in poor transport and storage capabilities and unacceptable organoleptic quality, while premature harvesting prevents the development of the characteristic flavor and aroma of tomatoes. 

During the ripening period, this fruit undergoes a number of physiological and biochemical processes, which change its chemical composition and cellular structure [2]. The ripening process of this fruit is linked with the degradation of chlorophyll and the accumulation of carotenoids in the fruit [3,4], changes in the aroma, degradation of cell wall polysaccharides which induces fruit softening [2,5], and autolysis of the locular tissues leading to the formation of a gel [6]. 

The texture, red peel color, characteristic flavor and aroma developed during this process have a significant effect on consumer approval, showing that tomato maturity is one of the most important factors associated with the quality of the final product [7,8,9]. Finding external factors associated with tomato maturity, and suitable for the precise assessment of the ripening stage has become a crucial issue in the crop industry. 

To date, tomato maturity has been monitored by means of simple surface color measurements [10], color measurements coupled with machine learning techniques [11], spatially offset Raman spectroscopy [12,13], evaluation of absorption and scattering coefficients measured by means of hyperspectral imaging systems [14], near infrared reflectance (NIR) spectroscopy [15], VIS-NIR spectroscopy [16,17] and the multi-parametric fluorescence techniques [18]. The relationship between internal structural changes and the stage of maturity of tomatoes was characterized by Zhang et al. [19] using magnetic resonance imaging and by means of X-ray computed tomography by Brecht et al. [6]. Mizrach [20] applied a nondestructive ultrasonic method to monitor the firmness and sugar content of greenhouse tomatoes. 

Although there are a number of possible methods, no standard for tomato maturity assessment has been provided to date. Externally measurable non-destructive factors associated with tomato maturity such as reflectance properties, color, size or shape, are not reliable due to the fact that they strongly depend on cultivar and agricultural conditions. Most of these methods cannot be applied in field conditions and may also be time consuming. 

Here we address this issue by applying the biospeckle method for monitoring tomato fruit maturation. Biospeckle is a dynamic interference pattern formed on a detector as a result of the backscattering of coherent light from living objects [21]. The dynamics of this phenomenon depend on the rate of metabolic processes inducing the micro movement of particles and organelles on a cellular scale [22,23]. In addition to this, biospeckle fluctuations also depend on the chlorophyll [24] and starch content [25], as well as the temperature of the plant sample under investigation [22].

At the present time, the literature describes a wide range of applications for dynamic laser speckle analysis. In common medical applications laser speckle technique helps to map a relative velocity of the moving cells or particles or in general monitoring of blood flow [26,27,28,29,30]. Recently Basak et al. [31] proposed an application of laser speckle contrast imaging for monitoring wound progression and characterization of cutaneous wound regions on mice model. Coherent light scattering speckle pattern analysis was also proposed as a diagnosis tool of occlusal caries lesions in deciduous molars [32]. Wang et al. [33] applied coherent light scattering for rapid and label-free recognition and classification of two cancer cell lines. Laser optical sensors were also used for screening and detection of bacterial colonies on agar plates [34] and screening of microbes in food [35]. Other application include structural mechanics [36], material sciences [37], studies of granular flow and paint drying [38,39].

The applications of the biospeckle technique in agriculture include the determination of the quality and the degree of maturation of fruits and vegetables [40,41,42,43], the analysis of seed viability [44], the detection of plant root bioactivity changes [45], the detection of fungal infection [40] or internal damage detection of fruit [46]. 

To date, only a few studies have addressed the monitoring of the ripening progress of tomatoes using the biospeckle method [47,48,49]. However, these studies considered application of the biospeckle to a limited extent, showing only the existence of temporal variations of biospeckle activity during the shelf-life, without comparison with the reference methods nor the bio-physical state of vegetables. 

This paper describes a shelf life experiment carried out with two tomato cultivars—Admiro and Starbuck. During shelf life storage tomatoes were monitored using a well-established optical method based on surface colour estimation and the biospeckle method. Both methods were tested with respect to their prediction capabilities of tomato maturity destructive indicators—firmness, chlorophyll and carotenoids content. This study aims to estimate the capabilities of the biospeckle method in terms of maturation monitoring and the prediction of the maturity indices of tomatoes.

## 2. Materials and Methods

### 2.1. Sample Material

In this experiment two tomato cultivars (*Solanum lycopersicum* L., cv. “Admiro” and “Starbuck”) were tested by means of nondestructive and destructive instrumental analysis. The fruit was obtained from the experimental fields of the Department of Vegetable and Medicinal Plants of The Warsaw University of Life Sciences. During the shelf-life experiment the tomatoes were stored for 11 days at an average humidity and temperature equal to 54.3 ± 6.4% and 27.1 ± 1.6 °C, respectively. During the whole shelf life experiment fifteen fruits from each cultivar were continuously monitored by means of non-destructive methods. Every day over the course of the experiment a batch of ten tomatoes was also sampled in order to perform destructive measurements. Therefore, in total, 250 tomatoes were used in this experiment.

### 2.2. Determination of Skin Color

The skin color of raw tomatoes was determined using the CM-5 Konica Minolta Chroma-Meter (Konica Minolta, Osaka, Japan). The measurements were performed in CIE L*a*b* color space. Samples were illuminated using a standardized source of diffused light D65, with a continuous spectrum similar to the daylight spectrum and a color temperature equal to 6505 K. The measurement area was equal to 8 mm. The readings of luminosity, a* and b* were taken from four sensing areas equally distributed at the equator of the fruit. From these values the chroma and color index were calculated. The color index was expressed as the a*/b* ratio, which represents the ratio of green-red to blue-yellow components of the skin color of the tomatoes. The chroma was defined as (a*^2^ + b*^2^)^1/2^. Finally, the chroma, a*/b* and luminosity values for each fruit were computed by means of four measurements.

### 2.3. Biospeckle Activity Video Sensor

**Figure 1:**
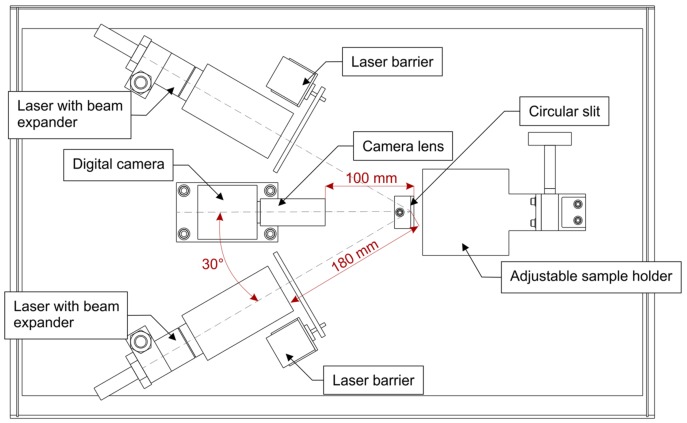
Schematically illustrated top view of the double wavelength biospeckle imaging system showing the location of system components.

In this study video sensor application software was deployed with double wavelength biospeckle imaging system illustrated schematically in Figure 1. System consisted two sources of laser light and digital camera as a light detector. Lasers worked independently, blocked by mechanical barriers so that only one laser illuminated the sample at the same time. The backscattered laser light from samples surface was recorded by a video camera. The programmable algorithm of a video sensor application software processed the image sequences, showing time evolution of granular patterns consisting of dark and bright spots, in order to evaluate the biospeckle activity of the sample. In this configuration system was evaluating biospeckle activity using a point sensing approach. 

The biospeckle activity was measured using two different light sources. It is a well-known fact that near infrared is a useful band for plant identification and phenotyping due to chlorophyll activity and absorption causing high reflectance in the NIR band [50]. Based on this the 830 nm LDM 830 laser (MONOCROM, Barcelona, Spain) with 30 mW of output power was chosen as the first light source. The second light source was chosen to match the spectral characteristic of ripening tomato which shows the highest changes between 550 and 700 nm with the broad peak around 675 nm corresponding to chlorophyll content [14]. With respect to this the 640 nm LDM 640 laser (MONOCROM) with 8 mW of output power was chosen as the second light source. In the case of both lasers the produced dots were too small and they were not able to illuminate the whole area under examination. Therefore, the laser beams were expanded by a 20× beam expanders (Edmund Optics GmbH, Karlsruhe, Germany).

The illumination angles for both lasers were equal to Θ ≈ 30°. Thins angle was chosen to reduce the amount of the specular light directly reflected from the tomato surface, which is undesired during observation the biospeckle phenomena. Lasers were placed about 180 mm from the tomato surface. This distance was chosen arbitrary as the smallest possible separation between light source and illuminated object in this imaging system (see Figure 1).

**Figure 2:**
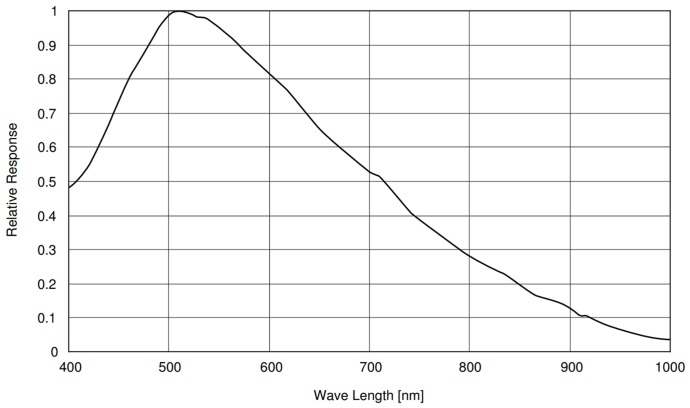
The relative response of the sensor in the visible and infrared spectrum.

The laser light scattered from the tomato surface was captured using a CCD camera (Monochrome FireWire Astronomy Camera DMK 21AF04.AS, The Imaging Source Europe GmbH, Bremen, Germany) with an ICX098BL sensor (Sony, Tokyo, Japan). The astronomy camera was chosen due to good low light sensitivity, which is a desirable feature in studies of the biospeckle phenomena. The resolution of monochrome sensor was equal to 640 × 480 (H × V) pixels with a 5.6 by 5.6 µm pixel size. The manufacturer provides a data sheet for this device, with additional specifications, namely: frame rate (up to 60 fps), sensor size (h × v = 4.6 × 3.97 mm), low light sensitivity (0.03 lux), type sensor format (1/4”), optical diagonal (4.5 mm), minimum/maximum exposure times (1/10.000 s to 30 s), gain and exposure modes (manual and auto), lens mount (C-Mount/CS), information about the operating temperature. The relative response of the sensor in the visible and infrared spectrum is showed in Figure 2. The camera was chosen to cover the wavelengths of both lasers. The camera had no built-in infrared filters. Since the relative response of the camera at 830 nm was lower than at 640 nm, then the output power of the 830 nm laser was chosen to be correspondingly higher compared to the 640 nm laser.

A small observation area allows uniform surface illumination and ensures that the influence of the surface curvature is negligible. Additionally, since the dynamics of the biospckle depend on the micro movement of particles and organelles on a cellular scale it was important to obtain a single pixel resolution smaller than the average cross section area of tomato pericarp cell. These conditions were ensured with the image resolution equal to 640 × 480 pixels and corresponding observation area of about 3 × 2 mm. The optical system was equipped with a lens with focal length of 25 mm, f-number varying from 1.4 to 16 covering maximum and minimum aperture respectively and format of 1/4” (Pentax Corporation, Tokyo, Japan). In the case of selected observation area, the working distance was shorter than the minimum object distance of the lens. Therefore, lens was attached to the camera via 20 mm extension ring in order to reduce the minimum object distance. With the extension ring the distance between the camera and the tomatoes was about 100 mm. The lens f-number was fixed to 16 (maximum value). Small aperture of the optical system is required to observe the biospeckle interference pattern. The smaller the aperture the bigger the dots of the interference pattern. In case of this study a special attention was taken to keep the size of recorded speckles no bigger than 3 × 3 pixels.

Biospeckle video sequences lasting 4 s were recorded in uncompressed AVI file format (8 bits, RGB24 codec) at a 15 frames per second rate. The exposition time was equal to 1/250 s and gamma was set to 100 (according to camera internal settings). Gain was set experimentally based on the histogram analysis to cover as much as possible of the dynamic range of the camera and avoid pixel over- and underexposure. The camera was connected with laptop via USB 2.0 communication interface. The laptop was running on Windows Vista 64-bit (Microsoft Corporation, Redmond, WA, USA) operating system. Video sequences were captured using the IC Capture 2.2 software (The Imaging Source Europe GmbH) provided by the camera manufacturer. The measurements were taken from four sensing areas equally distributed at the equator and averaged for each tomato.

**Figure 3:**
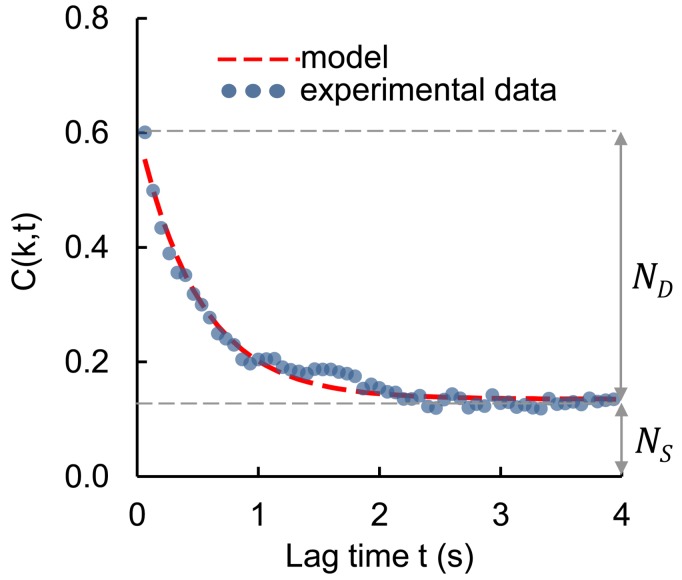
An example of time evolution of the *C(k,t)* value for *k =* 1, with an exponential decay function.

Based on the video stream data the video sensing application evaluated biospeckle activity using two separate measurements. Both were based on an analysis of the temporal changes of the biospeckle pattern. Time evolution of the biospeckle pattern may be described by the correlation coefficient *C(k,t)*, where k is the reference frame number and *t* is the lag time between the reference and referent frame. In practice, *C(k,t)* is calculated as the correlation coefficient of two data matrices, consisting of the intensities of pixels, captured with a lag of several seconds between them. Usually the first frame of the video sequence is chosen as the reference frame. Figure 3 shows an example of time evolution of the *C(k,t)* for *k =* 1. The temporal changes of the correlation coefficient can be modelled using the exponential decay function:

(1) $$C\left(k,t\right)=N_{D}e^{-at}+N_{s}$$

In this equation ***N_D_*** may be interpreted as the dynamic component of the biospeckle pattern (the portion of information stored in the biospeckle image that changes over time), while ***N_S_*** expresses the static component of this pattern (the information that is preserved over time). The *a* coefficient stands for the decorrelation constant, which defines the rate of change of the dynamic component of the biospeckle pattern. Finally, the first biospeckle activity measurement is defined as:

(2) $$\varepsilon =\frac{N_{D}}{N_{D}+N_{S}}$$

The second measurement C4 is simply defined as the *C(k,t)* value for *k =* 1 and *t =* 4 s. For a more meaningful representation the C4 coefficient is calculated as 1 − *C(k,t)* [22,24,25,40,43]. 

The C4 coefficient was chosen due to its simplicity, robustness and reliability which was showed in our previous studies. The parameter ε was chosen as an extension to C4, but calculated from larger data set. The C4 and ε can be successfully calculated on relatively slow computing units. Both methods have low computational and memory demands when compared to other methods such as LASCA, Fujii or Inertia Moment [44,51,52]. The calculation of C4 takes a fraction of a second and the measurement time is mainly influenced by the time interval between reference frames. For C4 the real time implementation can be easily accomplished. Calculation of ε takes considerably more time than C4, however with a few simple optimizations it can also be adapted to the real time implementations. The key advantage of C4 and ε, is that both methods measure how content of the biospeckle pattern changes with respect to reference frame in a given period of time. Contrary to this the intensity based methods (LASCA, Fujii or Inertia Moment) measure the amplitude of fluctuations of pixel intensity. Therefore, the intensity based methods are venerable to changes in image brightness. For instance LASCA is defined as the ratio of standard deviation to the mean value of the intensity of pixel fluctuations. This means that if the brightness of image increase without change in rate and amplitude of pixel intensity fluctuations (the same standard deviation), the apparent value of contrast will decrease. This leads to a conclusion that the value of intensity based methods can change without change in rate of observed process. Since in our experiment the luminosity of tomatoes surface was changing during the shelf life we decided to use C4 and ε as reliable indicators of biospeckle activity.

### 2.4. Firmness Test

The firmness of the tomatoes was measured by means of a standard puncture test, performed with a Texture Analyzer TA-TX2 (Stable Micro Systems Ltd., Surrey, UK; software: Texture Exponent 32, Stable Micro Systems Ltd.). The penetration depth was equal to 10 mm and a crosshead speed of 2 mm s^−1^ was used. Tests were carried out using a cylindrical probe with a diameter equal to 5 mm and a flat end. Firmness was determined as the maximum force registered during the test. Firmness was measured at the same locations where the skin color and biospeckle activity were determined.

### 2.5. Chlorophyll and Carotenoids Content

In order to evaluate the chlorophyll content tomatoes were peeled from skin and the seeds were removed. Samples of parenchyma tissue were taken only from the pericarp region of the fruit. Small pieces of the tissue were initially homogenized using a laboratory mill. Then, 50 mL centrifuge tubes with conical bottoms were filled with 3 g of homogenized tissue, 0.4 g MgCO_3_ and 15 mL of 80% acetone/water solution. This solution was homogenized for 1 min at 20,500 RPM (Yellowline D125 Basic, IKA, Wilmington, NC, USA). The homogenizer was washed using 80% acetone/water solution and the remnants were collected into test-tubes. The suspension was stirred using a laboratory stirrer at 2400 RPM for 1 min. The sediment was centrifuged at 6000 RPM (4428 *g*) for 10 min at 10 °C. The supernatant was poured into 25 mL measuring flasks and filled with 80% acetone/water solution. The absorbance of the supernatant was measured at 646 and 663 nm using a Helios v. 7.03 spectrophotometer (Thermo Electron Corporation, Waltham, MA USA) with the 80% acetone/water solution used as a reference sample. The total chlorophyll and yellow carotenoids content was estimated using the equations provided by Lichtenhaler and Wellburn [53] and expressed on a dried weight basis (g kg^−1^). The measurements of the yellow carotenoids content (mainly xanthophylls, without lycopene) were performed based on the absorbance at 470 nm. Taking into account the sample weight, volume of extract and dry matter content, results were expressed on a dried weight basis as mg kg^−1^. The chlorophyll and carotenoids content were measured in 5 replicates for representative samples taken from at least six different chopped tomatoes.

### 2.6. Statistical Analysis

The statistical significance of the daily changes in all of the measured parameters was tested using a one-way ANOVA test (using post hoc Tukey’s Honestly Significant Difference—HSD) for each cultivar independently. The correlation between biospeckle activity measurements and storage time was tested using standard linear regression models. Also, the correlation of daily averaged values of biospeckle activity measurements and optical parameters with firmness, chlorophyll and carotenoids content was tested using standard linear regression models. A statistical analysis of the experimental data was carried out using Statistica 10 software (StatSoft, Inc., Tulsa, OK, USA). A biospeckle analysis was carried out using own code developed in Matlab R2010a (MathWorks, Natick, MA, USA).

## 3. Results

### 3.1. Instrumental Analysis

**Figure 4:**
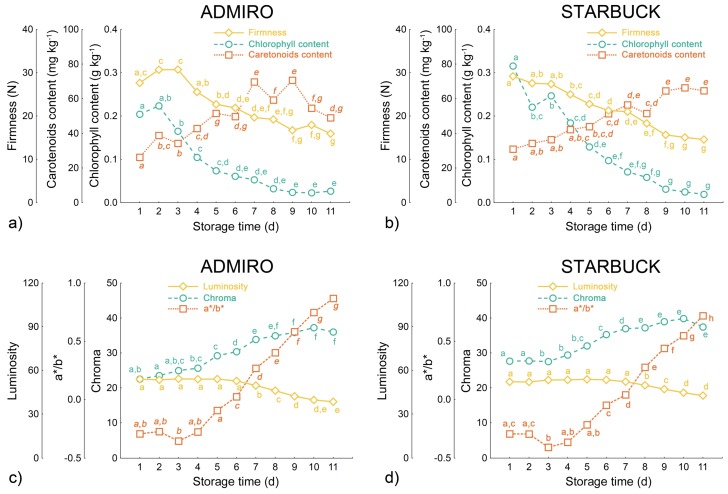
Daily averaged values of destructive maturity measurements (**a**,**b**) and the optical parameters of the tomato skin surface (**c**,**d**) for Admiro and Starbuck cultivars. Letter indexes indicate group membership according to a one-way ANOVA test with the *p* value equal to 0.05.

All physicochemical parameters measured during the shelf-life of the tested tomatoes showed considerable and significant changes associated with the ripening process. Figure 4 shows the changes in the mean values of these parameters with respect to the days of the shelf-life. A decrease in tomato firmness was observed during the period of shelf life storage. Fruit from both cultivars of tomato gradually softened starting from the first day of storage and continued to soften up to the last day. This was confirmed by the statistically significant differences in the average values of firmness. A similar trend was observed for the chlorophyll content. For both cultivars of tomato the chlorophyll content decreased starting from the first day of storage up to the 8th or 9th day when further changes become statistically insignificant. 

The carotenoids content showed the opposite relationship with respect to storage duration than the chlorophyll content. For both cultivars the average amount of carotenoids increased during the measured period of shelf life. However, measured changes were less consistent between the Admiro and Starbuck tomatoes. In the case of the Starbuck cultivar, the carotenoids content increased up to a maximum reached on the 9th day of storage (Figure 4b) just after the small but significant decrease on the 8th day. For the Admiro cultivar the maximum amount of carotenoids content was reached between the 7th and 9th day of shelf-life. Over the next two days the level of carotenoids decreased thus showing a statistically significant difference with respect to the previous maximum level. As was the case with the Starbuck cultivar, the Admiro tomatoes showed a slight but significant decrease in carotenoids levels on the 8th day of shelf-life with respect to the previous and the subsequent values. 

The optical parameters also showed considerable changes during storage, with a different level of sensitivity to the dynamics of the ripening process however. The luminosity for both cultivars was considered to be the least sensitive parameter. In both cases luminosity maintained its initial level during the first days of the experiment and then slowly decreased. The decrease became statistically significant starting from the 7th and 8th days for Admiro and Starbuck cultivars, respectively. Since the a*/b* ratio of the components of the CIE L*a*b* color space is associated with the green-red color range (it takes negative values for the green and positive for the red color) this parameter showed the highest variation in the average values during the shelf-life.

For both cultivars the a*/b* ratio started with a slight but not significant decrease in values after the first two days of shelf-life, maintaining its level up to the 6th day of storage, when both cultivars showed a significant increase in the values of this parameter. During the following days the a*/b* ratio showed a consistent increase in average values for both the Admiro and Starbuck cultivars. Similar trends were reported for the saturation levels which also showed a small but insignificant decrease on the last day of the experiment.

### 3.2. Biospeckle Analysis

**Figure 5:**
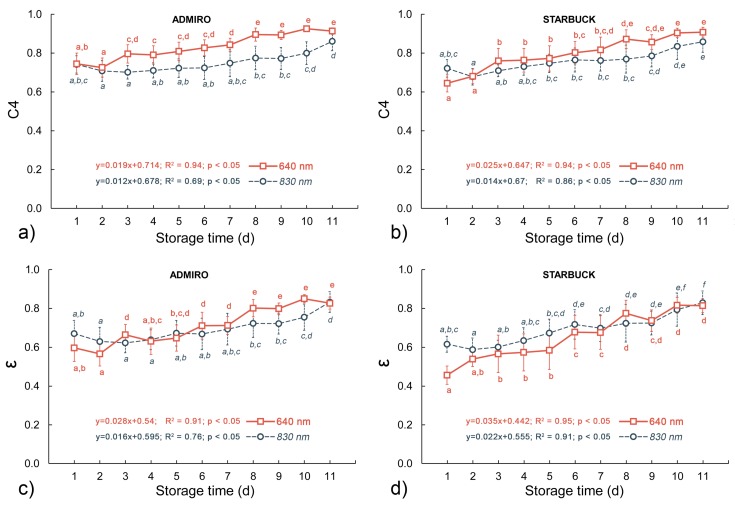
Changes in the mean values of biospeckle activity descriptors during shelf life storage for two laser wavelengths: (**a**,**c**) for C4 and ε for the Admiro cultivar, (**b**,**d**) for C4 and ε for the Starbuck cultivar. Letter indexes indicate group membership according to a one-way ANOVA test with the *p* value equal to 0.05, error bars show standard deviations.

The ongoing postharvest maturation of the tomatoes from both cultivars was clearly visible as a change in skin color from green to red. The postharvest maturation of tomatoes was tracked using two separate biospeckle activity measurements, obtained at two different laser light wavelengths. For both parameters the progressing maturation process corresponded with the increase in biospeckle activity. Figure 5 shows the biospeckle activity levels for both cultivars of tomatoes with respect to the days of the shelf life storage. 

For the C4 parameter the activity levels were higher for the 640 nm laser than for the 830 nm one. Also, the level of change between the first and the last day of the experiment was higher for this laser wavelength for both activity measurements. The ε values showed similar trends with respect to time of storage as the C4 values, however, there were smaller differences between activity levels at different laser wavelengths.

At the 640 nm laser wavelength the activity plateau was observed in the last days of the experiment, especially for the Admiro cultivar. This plateau was indicated by the lack of statistically significant differences between consecutive values of C4 and ε in the last four and two days of shelf life for the Admiro and Starbuck cultivars respectively. Nevertheless, the linear regression models showed significant and positive correlations with the time of storage for both biospeckle activity measurements at 640 nm. Both parameters showed close R^2^ values, with a slightly higher rate and range of change in the values for ε (as indicated by the coefficients of the linear regression models).

The biospeckle activity level captured using the 830 nm laser beam showed considerably lower variability in values than the corresponding parameters for the 640 nm wavelength. The lower range and rate of change was measured compared to the 640 nm beam, for both parameters and cultivars. Also, a slight decrease during the first days of shelf life was observed, which was then followed by a reversed trend. However, the positive and significant relationships of both biospeckle activity measurements with shelf life duration was still observed for the 830 nm laser beam wavelength. Comparing C4 and ε at 830 nm, the ε values showed a better correlation with storage time than the C4 values.

### 3.3. Postharvest Monitoring Using the Biospeckle Method

Standard linear regression models were built for daily averaged values of biospeckle activity measurements and optical parameters with firmness, chlorophyll and carotenoids content. Almost every model indicated the statistical significance of the tested relationships, however the quality of predictions varied with respect to the tomato cultivar, predicted parameter, laser wavelength etc. 

**Figure 6:**
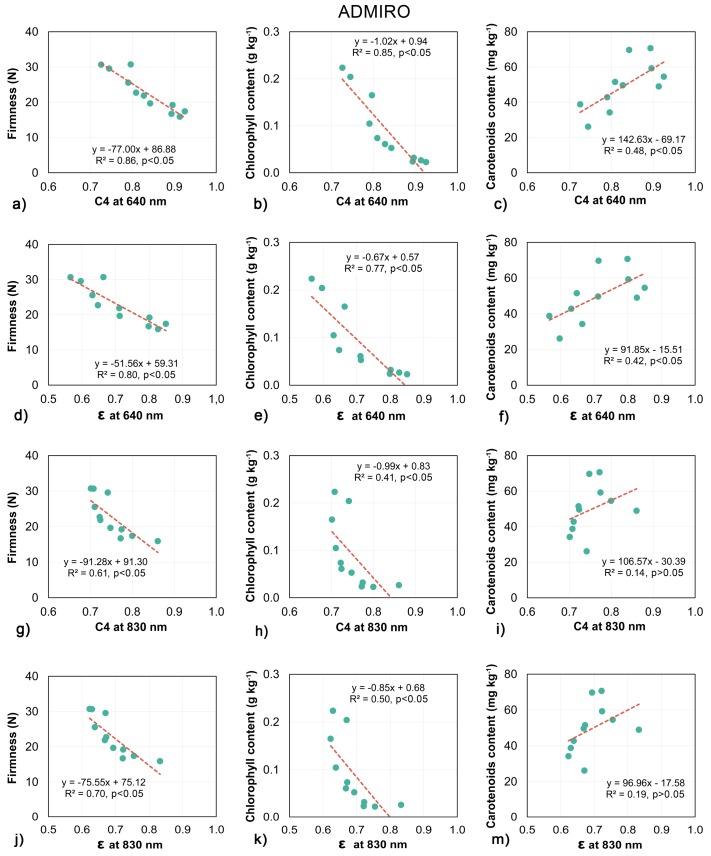
Linear regression models of the relationship between biospeckle activity descriptors and firmness (**a**,**d**,**g**,**j**), chlorophyll (**b**,**e**,**h**,**k**) and carotenoids content (**c**,**f**,**i**,**m**) for Admiro tomatoes cultivar.

**Figure 7:**
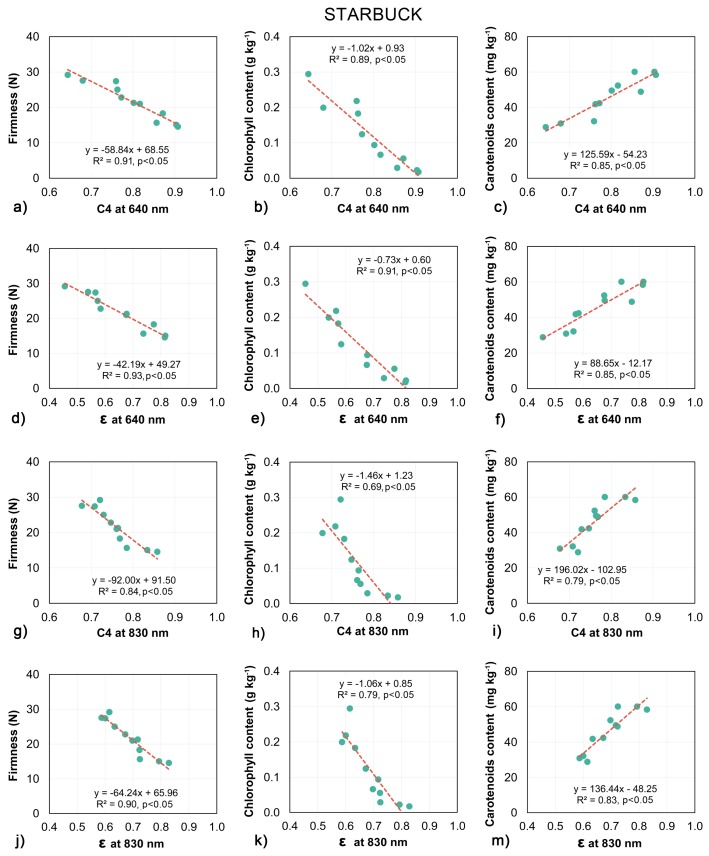
Linear regression models of the relationship between biospeckle activity and firmness (**a**,**d**,**g**,**j**), chlorophyll (**b**,**e**,**h**,**k**) and carotenoids content (**c**,**f**,**i**,**m**) for the Starbuck tomato cultivar.

The models created to examine the relationship between biospeckle activity and destructive measurements are shown in Figure 6 and Figure 7. In general, the biospeckle activity increased with the decrease in firmness and chlorophyll content, while increases in carotenoids content corresponded to an increase in biospeckle activity.

The biospeckle activity measured for both tomato cultivars showed a very high correlation with the firmness of the flesh of the fruit (Figure 6a,d,g,j and Figure 7a,d,g,j) The highest values in the determination coefficient for linear models were reported at 640 nm for Admiro and Starbuck tomatoes and at 830 nm for the Starbuck cultivar. Considerably lower performance was reported at the 830 nm wavelength (R^2^ = 0.61 for C4 and R^2^ = 0.70 for ε) for Admiro tomatoes. Also, the chlorophyll content was successfully predicted using linear models for both cultivars and both descriptors of the biospeckle activity. The biospeckle activity showed better prediction capabilities for the chlorophyll content using the 640 nm laser wavelength than the activity obtained with the 830 nm laser. This result held true for both tomato cultivars (Figure 6b,e,h,k and Figure 7b,e,h,k). 

The C4 and ε parameters obtained using the 640 nm laser showed a similar chlorophyll prediction performance, while at 830 nm slightly better results were achieved for the ε values. The worst results were reported for the Admiro cultivar at 830 nm where R^2^ values were equal to 0.41 and 0.5, for C4 and ε respectively.

The prediction performance of the carotenoids content varied significantly with respect to the cultivar of tomatoes (Figure 6c,f,i,m and Figure 7c,f,i,m). By far the worst predictions among all estimated parameters were obtained for the carotenoids content for the Admiro cultivar for both activity measurements and at both laser wavelengths. For this cultivar the correlations for C4 and ε at 640 nm were statistically significant but with only 50% of the predicted variance, while at the 830 nm laser wavelength no significant correlation was observed for both parameters. In contrast, every model examing the relationship between biospeckle activity and carotenoids content for the Starbuck cultivar showed that there were statistically significant, relatively high values of determination coefficients (from 0.79 for C4 at 830 nm to 0.85 for C4 and ε at 640 nm). The applied measurement of biospeckle activity results for both cultivars showed similar trends. At the 640 nm laser wavelength the prediction performance for C4 and ε was comparable, with slightly better results for C4. At the 830 nm wavelength a higher proportion of the predictable variance was obtained for the models created using the ε parameter. 

**Figure 8:**
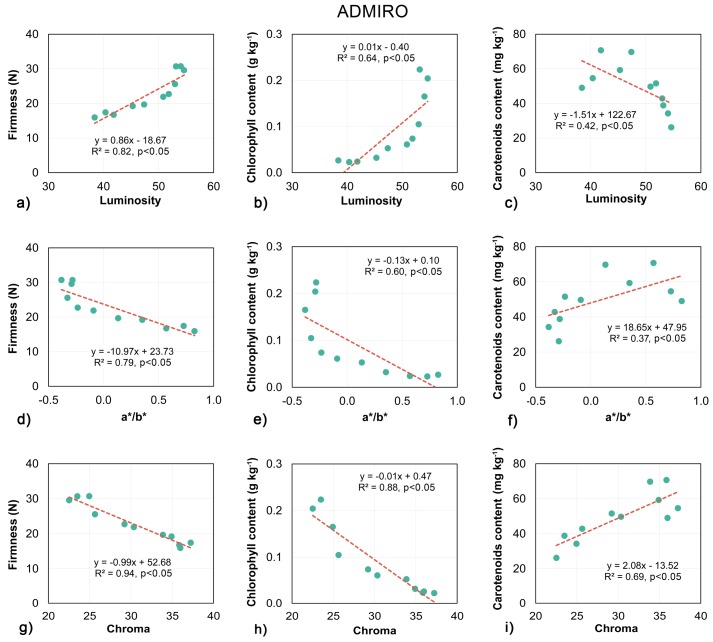
Linear regression models of the relationship between the luminosity (**a**–**c**), a*/b* ratio (**d**–**f**) and chroma (**g**–**i**) with firmness, chlorophyll and carotenoids content for the Admiro tomato cultivar.

**Figure 9:**
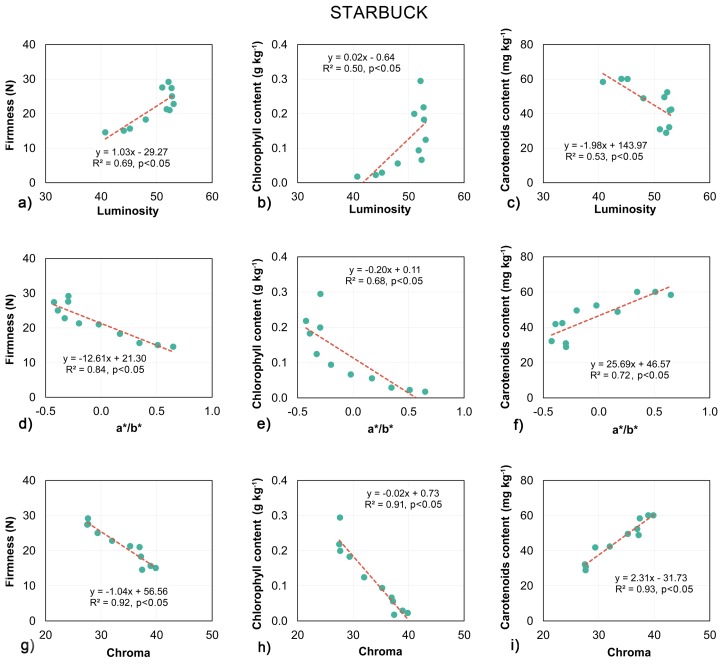
Linear regression models of the relationship between luminosity (**a**–**c**), a*/b* ratio (**d**–**f**) and chroma (**g**–**i**) with firmness, chlorophyll and carotenoids content for the Starbuck tomato cultivar.

The linear regression models for the reference optical parameters of the tomato surfaces are shown in Figure 8 and Figure 9. The results for both tomato cultivars showed similar trends in changes in firmness, chlorophyll and carotenoids content with respect to luminosity, a*/b* and chroma. The darkening of the skin was associated with the softening of the flesh. Also, the chlorophyll content decreased with the measured luminosity of the tomato surface. In contrast, the carotenoids content was negatively correlated with the luminosity (the darker the tomato the higher the carotenoids content measured). Both color associated measurements, namely chroma and a*/b* showed the opposite relationship. The values for both parameters increased with the decrease in firmness and chlorophyll content. The softening and degradation of the chlorophyll were associated with the transition in fruit skin color from green to red, and with an increase in the intensity of the coloring—chroma. Also, the higher the redness value and chroma of the tomatoes, the higher the carotenoids content which was measured. 

As with the biospeckle measurements, the optical parameters of the fruit skin showed high and significant correlations with the firmness of the tomatoes (Figure 8a,d,g and Figure 9a,d,g). The best predictions of firmness were obtained using chroma, with R^2^ equal to 0.94 and 0.92 for the Admiro and Starbuck cultivars respectively. Less satisfactory results were reported for luminosity and a*/b* with luminosity being more accurate for the Admiro and the a*/b* ratio being better in the case of the Starbuck cultivar. In contrast to this, the relationships reported between the luminosity and a*/b* and the chlorophyll and carotenoids content for both tomato cultivars were significant, however the R^2^ values showed very poor prediction capabilities (Figure 8b,e,h and Figure 9b,e,h). The results for these parameters varied from the worst predictions using a*/b* for the Admiro cultivar (R^2^ = 0.37) up to the best model obtained using the same parameter for the Starbuck cultivar (R^2^ = 0.72). The predictions of chlorophyll and carotenoids content using chroma showed very high accuracy (Figure 8 and Figure 9h,i) with R^2^ varying from 0.69 to 0.93. Comparing biospeckle analysis with the measurements of tomato skin optical parameters, the biospeckle approach was better at predicting firmness, chlorophyll and carotenoids content than luminosity and a*/b*. The biospeckle analysis showed similar or slightly lower performance when compared to the results obtained using chroma. The best overall results were obtained using chroma for both cultivars of tomatoes.

## 4. Discussion

Biospeckle analysis was tested as a potential tool for monitoring dynamic changes in the physiological features of tomatoes during postharvest ripening. The performance of biospeckle imaging was verified against standard optical measurements (chroma, a*/b* ratio and luminosity) in predictions of the firmness and changes in the chlorophyll and carotenoids content. The relationship of the tomato skin parameters and the biospeckle activity with the destructive indicators of maturity was investigated using linear regression models.

The choice of both laser light wavelengths for this study was motivated by the specific optical features of tomato pericarp tissue constituents and their changes during maturation. Zhu et al. [14] measured the optical absorption and scattering properties of tomatoes during ripening. The absorption profiles in the range of 550–700 nm showed a typical pattern that reflected the gradual loss of chlorophyll and accumulation of carotenoids during ripening. The broad peak around 675 nm corresponding to chlorophyll content decreased consistently with the increasing ripeness grade reaching a minimum for red tomatoes. At the same time the overall absorption level in the spectral region of 550–600 nm showed an increase indicating the accumulation of carotenoids. It was also demonstrated that tomatoes had near zero absorption over the wavelength region of 700–900 nm. In this region the optical properties of tomato flesh were dominated by the scattering coefficient value. At the early ripeness stages the scattering coefficient value decreased, while for late ripeness grades this trend was reversed. This was attributed first to cell wall depolymerisation and cellulose degradation and later to the production of small pigment molecules and soluble pectin that increased the density of the tomatoes. 

All of this suggests that the light generated at two different laser wavelengths used in this study should have been altered by different underlying phenomena and thus should have different prediction capabilities with respect to the measured physio-chemical properties. Since the photon transfer of laser light at 640 nm was mainly influenced by the absorption properties of chlorophyll and carotenoids, the biospeckle activity for this wavelength should be more sensitive to the chemical changes in tomatoes. In contrast to this, the biospeckle activity at the 830 nm laser wavelength should indicate structural changes such as cellular structure degradation, the amount and size of the particles, as well as the velocity of their movement, which mainly influence the scattering properties of tomato tissue.

This could partially explain the changes in the biospeckle activity reported in this study for both tomato cultivars at the 830 nm of laser light wavelength. The values of both activity measurements slightly decreased during the first days of shelf-life and then showed a gradual increase (Figure 5a and Figure 4b). Although, the initial decrease was not statistically significant, the increase in the following days was, showing a similar trend to the scattering coefficient changes reported by Zhu et al. [14]. Another possible explanation is that the biospeckle activity reflects the chlorophyll and carotenoids content relationship. Figure 4a,b show that the greatest falls in cholorphyll content occurred during the first days of storage, and later on were followed by a gradual increase in the amount of carotenoids.

Moreover, the spectrodiometric measurements of tomato pericarp tissue showed that during transition from the immature-green to the turning stage, the amount of red light that passed through these tissues increased 4-fold [54]. At the same time the level of far-red light changed very little. Further maturation showed no effect on the transmission of red and far-red light.

This observation corresponds very well with the current study, as the greater penetration depth allowed the laser light to reach more scattering surfaces at the 640 nm wavelength, this resulted in higher C4 values for the 640 nm wavelength than for the 830 nm wavelength. No further changes in the transmission properties of the tissue would result in a lower rate of change in biospeckle activity; in this study that scenario was described as the biospeckle activity plateau. However, it should also be noted that C4 showed relatively high values even at the very beginning of the experiment, and approached its maximum possible level in the following days of storage. At such high levels of activity the C4 coefficient loses its sensitivity and resolution. Therefore, a shorter decorrelation lag time between frames should be considered to increase the performance of the biospeckle method at late maturation stages.

Carotenoids and chlorophyll are considered to be a good maturity indicator since their content changes significantly during the ripening process. In this study carotenoids were found in tomato tissues at the very beginning of the shelf life experiment confirming that they were harvested at the mature green stage. During the following days of maturation the total carotenoids content gradually increased, doubling its initial value at the 11th day of storage (Figure 4a,b). Similar trends were reported by other researchers [55].

Qin et al. [13] characterized the carotenoids development pattern during the ripening of tomatoes with respect to their localisation inside the fruit. It was reported that at the immature green stage of development of the tomatoes the Raman chemical images of the sliced fruit did not show any carotenoids. The mature green tomatoes also showed no carotenoids in the outer pericarp but did show the initial appearance of these compounds in the locular tissues. As the tomatoes ripened, the carotenoids content increased, reaching a maximum level for fully ripened red fruit. Considering that for soft tissues laser light has strong penetration abilities one could assume that the biospeckle method will be able to detect changes in the amount of subsurface carotenoids at the early stage of maturity of tomatoes.

However, the biospeckle analysis demonstrated a rather moderate performance in predicting the amount of subsurface carotenoids. For the Admiro cultivar both biospeckle activity measurements at 640 nm and 830 nm wavelengths were unable to reflect the changes in carotenoids content (Figure 6c,f,i,m) and no statistically significant relationship was found. In contrast to this situation for the Starbuck cultivar the C4 and ε parameters showed high positive and significant correlations with changes in the carotenoids content for both laser wavelengths.

Since carotenoids are the dominant pigments responsible for tomato skin colour, it was to be expected that their content would be correlated with values of luminosity, chroma and especially the a*/b* ratio. Other studies for tomatoes have shown that the accumulation of lycopene accelerates from the pink stage and there is a high correlation between lycopene content and color values (a*/b*). The higher the ratio of a*/b*, the higher the lycopene content [56]. Arias et al. also measured tomato lycopene content using HPLC, and then correlated these results with color measurements [57]. The a, a*/b* color factors produced the best regressions. Similar results were also reported for other fruits such as pumpkins where the a*, b* and chroma values were highly correlated with the total carotenoids content [58]. However, in this study surprisingly poor results were reported for a*/b* and luminosity for the prediction of carotenoids content. Both parameters failed in the case of Admiro tomatoes, with a moderate performance of the a*/b* ratio only achieved the Starbuck cultivar (Figure 9f). The best results were obtained using chroma which for both cultivars demonstrated more accurate predictions than both of the biospeckle activity descriptors.

It should be stressed that in this study the measurements of total carotenoids content were carried out with respect to just the yellow carotenoids. Therefore, these measurements did not fully reflect the chemical changes occurring during tomato maturation. This is probably the main reason for the low prediction performance of the biospeckle method and reference measurements with respect to this parameter. 

Compared with the carotenoids content, the results obtained for chlorophyll showed a more pronounced relationship with biospeckle activity. The highest correlations were reported for the 640 nm laser wavelength for the Starbuck and Admiro cultivar (Figure 6b,e and Figure 7b,e). Better results for the Starbuck cultivar were attributed to a more stable and closer to linear decrease in chlorophyll content, whereas in the case of the Admiro tomatoes chlorophyll levels decreased rapidly in the first four days of shelf-life and then slowly approached the minimum level (Figure 4a,b). This is clearly visible during the late maturation stages when the values of C4 and ε are still increasing (Figure 5), while the chlorophyll content shows little change. The better prediction results for the 640 nm laser over the 830 nm version are not surprising since the red laser wavelength falls into the chlorophyll absorption spectrum. The experiments have shown that, for apples, and probably for any materials containing chlorophyll, biospeckle activity is linearly masked by chlorophyll light absorption when red lasers are used. This effect results in the underestimation of particle activity within cells when the biospeckle method is used [24]. This phenomena was confirmed by the very low sensitivity of the biospeckle analysis to changes in the chlorophyll content at the 830 nm laser wavelength (Figure 6h,k and Figure 7h,k). Especially for the Admiro cultivar it may be observed that when the chlorophyll content decreases three times from its initial level the biospeckle descriptors increases only a little (Figure 6h,k). However, it should be noted that avoiding the masking effect did not result in a better prediction of performance for other parameters at this wavelength (firmness and carotenoids content). This could be attributed to the changes in the scattering properties of tomatoes during ripening, mentioned earlier. The results for reference parameters a*/b* and luminosity again demonstrated very low prediction capabilities with low sensitivity to large variations in chlorophyll content (Figure 8b,e and Figure 9b,e). As with the carotenoids, very accurate predictions of the chlorophyll content for both cultivars were provided by means of the chroma parameter. These results were also comparable with the performance of the biospeckle method using the 640 nm laser beam.

Firmness is one of the main indicators of tomato quality which determine consumer acceptability. In this study, tomatoes showed a characteristic decrease in firmness which occurred during shelf life maturation [10,16,17]. This behaviour could be partially attributed to weight loss due to water evaporation and thus a decrease in turgor pressure [59,60], but mainly it was induced by the deterioration of the cell wall structure [5]. Cybulska et al. [61] and Zdunek et al. [62] demonstrated that for various fruits and vegetables pectin enzymatic degradation during ripening, which occurs both in the network and to individual molecules, is one of the mechanisms responsible for softening. Less degraded, thicker and more branched pectin molecules were associated with better firmness and a more favourable texture. In the same way, the tomato fruit softening process involves the enzymatic degradation of cell wall polysaccharides (pectin) linked with pectin esterase and polygalacturonase enzyme activity. Based on atomic force microscopy analysis Xin et al. [63] demonstrated that the pectin polymers of ripe tomatoes degraded more with lower firmness than unripe tomatoes and that the percentage of wide chains decreased during ripening. Cell wall depolymerisation and the increased solubility of the middle lamella results in an increase in the number of smaller particles with a higher surface area to volume ratio than the large ones. With more scattering surfaces per unit mass than large particles, small particles directly impact the scattering properties of tomato tissue [14]. This hypothesis was very well supported by the predicted performance of models showing the relationship between biospeckle activity and firmness (Figure 6a,d,g,j and Figure 7a,d,g,j). High and significant correlations were observed for both cultivars with a similar performance at 640 nm and 830 nm wavelengths. A slightly lower performance was reported for the Admiro cultivar at 830 nm with R^2^ equal to 0.61 and 0.7 for C4 and ε respectively. Apart from the very favourable results for biospeckle activity descriptors, firmness was also successfully predicted using all non-destructive optical parameters. 

## 5. Conclusions

The biospeckle method demonstrated very good results in terms of maturation monitoring and in the prediction of the maturity indices of tomatoes, proving the possibility of the practical implementation of this method for the determination of the maturity stage of tomatoes and thus defining the optimal harvest window. The performance of the biospeckle method varied with respect to the tomato cultivar used and the applied wavelength of laser light. For both wavelengths the climacteric maturation of the tomato fruit corresponded to an increase in biospeckle activity. In general, the biospeckle activity captured at 640 nm demonstrated a higher range of changes (higher sensitivity) and a higher correlation with shelf life storage time than the corresponding measurements using the 830 nm laser beam. The biospeckle activity measured at 640 nm also enabled more accurate predictions of firmness, chlorophyll and carotenoids content than the 830 nm laser wavelength. At the 640 nm laser wavelength both activity descriptors (C4 and ε) provided similar results, while at the 830 nm wavelength the ε value demonstrated a slightly better performance. The best performance of the biospeckle analysis method was achieved for the firmness of tomatoes using both laser wavelengths. Lower prediction accuracy was obtained for the chlorophyll content using the 830 nm laser beam to investigate the properties of the Admiro cultivar. Rather moderate accuracy of the biospeckle method was reported for the carotenoids content. For the Admiro cultivar both bioscpeckle activity indicators showed no correlation with the carotenoids content, while for the Starbuck tomatoes the linear regression models showed significant relationships with relatively high R^2^ values. Compared to the standard optical measurements the linear regression models demonstrated that biospeckle activity descriptors had a higher correlation with chlorophyll and carotenoids content than the a*/b* ratio and luminosity. Among all of the tested optical parameters chroma demonstrated the best performance in terms of the prediction of firmness, chlorophyll and carotenoids content. Moreover, the results for chroma were comparable with the results for both biospeckle activity indicators. Compared to chlorophyll and carotenoids content, firmness was predicted with the highest accuracy using the biospckle approach as well as standard optical measurements. Further studies should focus on building more reliable and accurate models that make use of a larger dataset. High values of C4 indicated that with the applied acquisition settings the biospeckle analysis was near the detection limits of the dynamics of changes in the biospeckle pattern, therefore an optimisation of the biospeckle processing method is needed.
